# Perioperative Search for Circulating Tumor Cells in Patients Undergoing Prostate Brachytherapy for Clinically Nonmetastatic Prostate Cancer

**DOI:** 10.3390/ijms18010128

**Published:** 2017-01-11

**Authors:** Hideyasu Tsumura, Takefumi Satoh, Hiromichi Ishiyama, Ken-ichi Tabata, Kouji Takenaka, Akane Sekiguchi, Masaki Nakamura, Masashi Kitano, Kazushige Hayakawa, Masatsugu Iwamura

**Affiliations:** 1 Department of Urology, Kitasato University School of Medicine, Sagamihara 252-0374, Japan; tsatoh@kitasato-u.ac.jp (T.S.); ktabata@med.kitasato-u.ac.jp (K.T.); miwamura@med.kitasato-u.ac.jp (M.I.); 2 Department of Radiology and Radiation Oncology, Kitasato University School of Medicine, Sagamihara 252-0374, Japan; hishiyam@kitasato-u.ac.jp (H.I.); takenaka@kitasato-u.ac.jp (K.T.); akane.o.enaka@gmail.com (A.S.); m-kitano@jcom.home.ne.jp (M.K.); hayakazu@med.kitasato-u.ac.jp (K.H.); 3 Department of Microbiology, Kitasato University School of Allied Health Sciences, Kanagawa 252-0373, Japan; nakamu7@mac.com

**Keywords:** prostate cancer, brachytherapy, circulating tumor cell

## Abstract

Despite the absence of local prostate cancer recurrence, some patients develop distant metastases after prostate brachytherapy. We evaluate whether prostate brachytherapy procedures have a potential risk for hematogenous spillage of prostate cancer cells. Fifty-nine patients who were undergoing high-dose-rate (HDR) or low-dose-rate (LDR) brachytherapy participated in this prospective study. Thirty patients with high-risk or locally advanced cancer were treated with HDR brachytherapy after neoadjuvant androgen deprivation therapy (ADT). Twenty-nine patients with clinically localized cancer were treated with LDR brachytherapy without neoadjuvant ADT. Samples of peripheral blood were drawn in the operating room before insertion of needles (preoperative) and again immediately after the surgical manipulation (intraoperative). Blood samples of 7.5 mL were analyzed for circulating tumor cells (CTCs) using the CellSearch System. While no preoperative samples showed CTCs (0%), they were detected in intraoperative samples in 7 of the 59 patients (11.8%; preoperative vs. intraoperative, *p* = 0.012). Positive CTC status did not correlate with perioperative variables, including prostate-specific antigen (PSA) at diagnosis, use of neoadjuvant ADT, type of brachytherapy, Gleason score, and biopsy positive core rate. We detected CTCs from samples immediately after the surgical manipulation. Further study is needed to evaluate whether those CTCs actually can survive and proliferate at distant sites.

## 1. Introduction

Brachytherapy approaches have been accepted as a useful method to control localized and locally advanced prostate cancers [1,2,3,4,5]. One of the most appealing reasons for selecting this treatment is favorable long-term outcome with a low degree of toxicity [6]. Low-dose-rate (LDR) brachytherapy provides superior outcomes in patients with low- and intermediate-risk diseases [1,2]. The combination of high-dose-rate (HDR) brachytherapy and external irradiation is an effective treatment for delivering radiation doses more precisely in prostate cancer, even if patients have extracapsular invasion and seminal vesicle invasion [3,4,5]. Technical modifications for prostate brachytherapy are being developed to obtain the better treatment outcome [2,7,8,9,10]. However, approximately 5%–20% of those patients, as it now stands, show recurrence within 5 years after brachytherapy [1,2,5,11,12].

When patients are suspected to have treatment failure, evaluation—including abdominal computed tomography scan, pelvic magnetic resonance imaging, a bone scan, and prostate biopsy—are usually conducted to identify the site of relapse. Some patients develop distant metastases despite the absence of local recurrence. In those cases, micrometastasis that was not detected by radiographic images may have been present at initial diagnosis. Another possibility is that surgical manipulation that involves needles being inserted into prostate tissue may pose a potential risk for hematogenous spillage of prostate cancer cells and play a role in distant metastases in patients undergoing prostate brachytherapy. A no-touch isolation technique, in which vascular control is achieved prior to tumor manipulation, is generally considered to reduce cancer dissemination and subsequently reduce future disease recurrence in various cancer-related surgeries [13,14,15,16,17]. However, this technique is not used during brachytherapy procedures. In addition, needles being inserted into prostate tissue directly penetrate the cancer lesions at a certain rate.

Elucidating the mechanism and causes of relapse is a key challenge for the enhancement of treatment outcome [18,19]. We suspect that iatrogenic circulating tumor cell (CTC) spillage can convert a nonmetastatic cancer to a systemic one. In this study, we evaluated whether brachytherapy procedures can provoke hematogenous spillage of prostate cancer cells. We detected perioperative CTCs using the CellSearch System and compared preoperative CTC counts with intraoperative ones. We analyzed whether intraoperative CTC increases were associated with perioperative clinicopathological features.

## 2. Results

**Figure 1:**
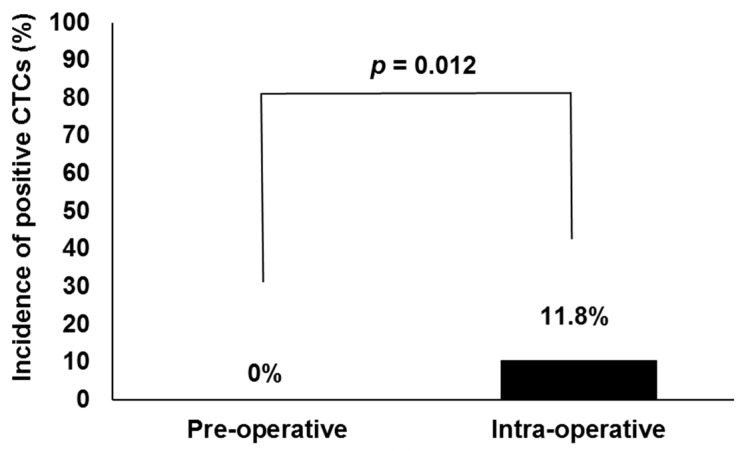
Comparison of circulating tumor cell (CTC) detection rates between pre- and intraoperative blood specimens in all patients undergoing high-dose-rate or low-dose-rate brachytherapy (*n* = 59).

Characteristics of the 59 patients are shown in Table 1. As shown in Figure 1, no CTCs were detected in preoperative samples. CTCs were detected from samples collected immediately after insertion of needles in 7 of 59 patients (11.8%). Intraoperative CTC detection rates were significantly higher than preoperative ones (11.8% vs. 0%, *p* = 0.012).

**Figure 2:**
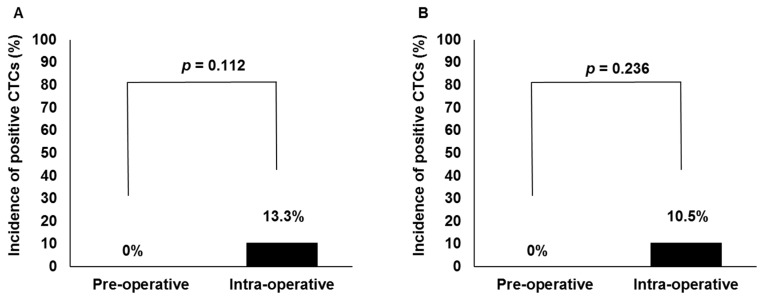
Comparison of circulating tumor cell (CTC) detection rates between pre- and intraoperative blood specimens in patients undergoing high-dose-rate (**A**, *n* = 30) and low-dose-rate (**B**, *n* = 29) brachytherapy.

Figure 2A,B showed perioperative CTC detection rates in patients undergoing HDR and LDR brachytherapy, respectively. Intraoperative CTCs were detected in 4 of 30 (13.3%) patients and 3 of 29 (10.5%) patients with HDR and LDR brachytherapy, respectively. While intraoperative CTC detection rates were relatively high when compared with preoperative ones in each group, according to a Fisher’s exact test, the differences did not reach statistical significance in the HDR brachytherapy group (*p* = 0.112) or in the LDR brachytherapy group (*p* = 0.236).

Table 2 lists the characteristics of the 7 patients who became positive for CTCs intraoperatively. The intraoperative CTC count was 1 CTC in 3 patients and 2 CTCs in 1 patient treated with HDR brachytherapy, and 1 CTC was detected in 3 patients who underwent LDR brachytherapy.

To investigate the secondary outcome measures, patients were divided into two groups according to positive or negative status for intraoperative CTCs. Positive status did not correlate with clinicopathological and perioperative variables, including use of neoadjuvant hormonal therapy, type of brachytherapy, age, prostate-specific antigen (PSA) at diagnosis, Gleason score, clinical stage, biopsy positive core rates, prostate volume at brachytherapy, or National Comprehensive Cancer Network (NCCN) risk criteria 2015 (Table 3). Neither patients with positive status nor those with negative status for intraoperative CTCs had postoperative clinical progression, with a median follow-up of 18 months (range, 15–24 months).

## 3. Discussion

In this study of clinically nonmetastatic prostate cancer patients, we detected CTCs from samples immediately after insertion of needles in patients undergoing prostate brachytherapy. Intraoperative CTC detection rates were significantly higher than preoperative ones. Our results may support a potential risk for hematogenous spread of cancer cells during the procedure.

Historically, transurethral resection of prostate (TURP) was generally performed to relieve the urinary tract obstruction caused by prostate cancer. In 1986, Levine et al. reported the possibility that cancer cells might be disseminated during TURP in patients with clinically evident cancer confined to the prostate [13]. They noted that the 5-year survival rate in those patients undergoing TURP was significantly lower than those not undergoing the procedure (*p* = 0.02). Several investigators then measured perioperative CTCs and reported the possibility of hematogenous spillage of cancer cells during radical prostatectomy in clinically nonmetastatic cancer patients [20,21,22,23,24]. Eschwege et al. investigated the dissemination of malignant prostatic cells during open radical prostatectomy [20], and they confirmed prostate-specific membrane antigen (PSMA) using reverse-transcription nested PCR for CTC detection. The incidence of positive CTC status increased from 21% before the surgery to 86% immediately afterward, supporting the possibility of intraoperative hematogenous dissemination during the open radical prostatectomy. They concluded that surgeons should minimize prostate manipulation to avoid seeding from the gland for the prevention of metastatic disease.

Prostate needle biopsy is one of the most similar procedures to prostate brachytherapy in that needles being inserted into prostate tissue directly penetrate the cancer lesions. Hara et al. examined PSA-mRNA-bearing cells in peripheral blood of the 108 patients before and after prostate biopsy [25]. Of 46 patients who were diagnosed with prostate cancer, the incidence of positive PSA-mRNA-bearing cells increased from 3% before the biopsy to 45% immediately afterward. In addition, the incidence of positive PSA-mRNA status after prostate biopsy in patients diagnosed with prostate cancer were higher than those without prostate cancer (45% vs. 25%, *p* < 0.001). This study supported the possibility of tumor spreading by prostate biopsy.

While we detected intraoperative hematogenous spillage of prostate cancer cells during brachytherapy procedures, it is still controversial whether the CTCs spilled iatrogenically into circulation have the biological capability to implant into distant sites and subsequently develop metastatic foci. Most studies—including the present study that detected the intraoperative CTC increase during radical treatment for primary lesion and prostate biopsy—had a small sample size and lacked a long follow-up period. Thus, the clinical significance of intraoperative CTC increase remains unclear, and we still have the question of whether this kind of iatrogenic CTC is clinically metastable. Eschwege et al. evaluated the cancer-cell seeding impact on recurrence-free survival [23]. Hematogenous spread of prostate cells was assessed by a dual PSA/PSMA PCR assay using very specific PSMA and PSA primers. Ninety-eight patients with negative status for preoperative CTC were divided into two groups according to status for intraoperative CTC: 53 (54%) remained negative and 45 (46%) became positive. Median biological and clinical recurrence-free time did not significantly differ between the two groups (69.6 vs. 65 months). The authors concluded that intraoperative hematogenous spillage of prostate cancer cells does not have a statistically significant adverse effect on recurrence. Their results seem to exclude tumor surgical management as a major cause of metastatic development. Some studies demonstrated that a primary tumor contains subpopulations of metastatic and nonmetastatic cancer cells. Only a restricted fraction of the cells in a primary tumor are considered to be highly metastatic [26,27]. Mareel et al. reported that 0.1% of CTC is responsible for the formation of metastatic foci [28]. When intraoperative hematogenous spillage of prostate cancer cells occurs during surgical procedures, we suspect that it may provoke distant metastases. However, the possibility of metastatic foci formation caused by the iatrogenic CTC spillage from a primary tumor may occur fairly infrequently.

We could not find any association of intraoperative CTC increases with perioperative clinicopathological features in the present study. This may reflect the fact that all patients undergoing brachytherapy have a risk of intraoperative hematogenous spillage of prostate cancer cells, irrespective of use of neoadjuvant hormonal therapy, type of brachytherapy, age, PSA at diagnosis, Gleason score, clinical stage, and biopsy positive core rates. In our regiment of HDR brachytherapy for high-risk and locally advanced cancers, we administered at least 6 months of neoadjuvant androgen deprivation therapy (ADT). Nonetheless, intraoperative CTCs were detected in 13.3% of patients in that group. Although the use of neoadjuvant ADT may reduce the cellular viability of iatrogenic CTCs enough to prevent implantation into distant sites, this could not completely eliminate the hematogenous spillage of CTCs during the procedure.

Several potential limitations of this study must be considered. The sample size for the present study was not calculated to detect a statistical difference in clinical progression between patients with intraoperative CTC increases and those without increases. A large-scale study involving more patients is needed to clarify whether intraoperative CTC increases actually affect the postoperative progression. Second, samples for CTC detection were not drawn before neoadjuvant ADT in patients treated with HDR brachytherapy. These patients were classified with clinically high-risk or locally advanced cancer and were more likely to have occult distant metastasis than lower-risk patients. Some of these patients may have had positive status for CTC before neoadjuvant ADT [29]. In addition, samples for CTC detection were not drawn a few days or months after the brachytherapy procedures in patients with positive status for intraoperative CTCs [20]. Longer detection of CTCs may have a higher risk of later metastases than others. Third, the CellSearch System may lack sensitivity in nonmetastatic cancer patients and consequently underestimates the incidence of perioperative CTCs. In addition, this system only detects the epithelial cancer cells and does not detect the mesenchymal ones. In metastatic formation from primary epithelial cancers, epithelial–mesenchymal transition at primary sites is considered to be important for cancer metastasis [30,31]. This cellular transition allows epithelial cancer cells to acquire the more invasive characteristics. The mesenchymal cancer cells may have a higher metastatic potential than the epithelial ones. In future clinical series, the detection of such highly metastatic potential cells may be helpful in assessing the possibility of metastatic diseases caused by iatrogenic CTCs during prostate brachytherapy procedures.

## 4. Materials and Methods

### 4.1. Patient Selection

From October 2014 to July 2015, 59 patients with clinically nonmetastatic prostate cancer who underwent HDR or LDR brachytherapy participated in this prospective study. Thirty patients with high-risk or locally advanced prostate cancer were treated with HDR brachytherapy. Twenty-nine patients with clinically localized prostate cancer were treated with LDR brachytherapy. Samples of peripheral blood were drawn before insertion of needles (preoperative) and again immediately after the surgical manipulation (intraoperative) in each patient. Pretreatment evaluation included clinical history, physical examination, blood laboratory findings, pelvic computed tomography, pelvic magnetic resonance imaging, and a bone scan. Exclusion criteria were a history of cancer, previous surgery for benign prostatic hyperplasia, or concomitant active urinary tract infection. Patients who had a suspicious lesion of cancer other than prostate cancer on the pretreatment evaluation were also excluded. Patients were removed from the study if they wished to discontinue. All biopsy slides were reviewed by our institutional pathologists. Approval was granted by the ethics committee of our institution (B14-20), and all patients signed written informed consent.

### 4.2. HDR Brachytherapy and Blood Sample Collection

The protocol and procedure for HDR brachytherapy and hormonal therapy in high-risk or locally advanced prostate cancer were reported previously [5,32]. All patients underwent ≥6 months of neoadjuvant ADT, which combined nonsteroidal anti-androgen agents with luteinizing hormone-releasing hormone agonist injections. Either flutamide (375 mg/day) or bicalutamide (80 mg/day) were prescribed as the nonsteroidal anti-androgen agents. Either goserelin (10.8 mg/3 months or 3.6 mg/month) or leuprorelin (11.25 mg/3 months or 3.75 mg/month) were administrated as luteinizing hormone-releasing hormone agonist therapy.

In the operating room, preoperative samples of peripheral blood were drawn in a supine position before epidural anesthesia. Patients were then placed in a lithotomy position. Metallic marker seeds were placed transperineally into the base and apex for the purpose of image-guided external beam radiotherapy following HDR brachytherapy. Treatment was started using placement of a closed transperineal hollow needle under transrectal ultrasound guidance. Multiple 25 cm long, closed-end, 15-G plastic hollow needles were inserted transperineally using a 15-G Prostate Template (Best Medical International Inc., Springfield, VA, USA). Eighteen needles were routinely implanted. Flexible cystoscopy was conducted to check that the urethra had not been penetrated by the implanted needles. The needle tips were left within the urinary bladder, 15 mm above the sonographically or cystoscopically defined base of the prostate. Immediately after all of these procedures had been completed, intraoperative samples of peripheral blood were drawn from each patient again.

### 4.3. LDR Brachytherapy and Blood Sample Collection

The protocol and procedure for LDR brachytherapy were performed as previously reported [8]. No patients were treated with ADT and external beam radiation therapy before and after LDR brachytherapy.

In the operating room, preoperative samples of peripheral blood were drawn in a supine position before spinal anesthesia. Patients were then placed in a lithotomy position. Results of transrectal ultrasonography in the axial plane were imported into the VariSeed brachytherapy planning system (Varian Medical Systems, Palo Alto, CA, USA). The prostate, urethra, and rectal wall were contoured by radiation oncologists. Seed number and location for both peripheral and centrally located needles were determined manually. The prescribed dose was set at 145 Gy. Dose–volume histograms and isodose lines were evaluated based on predetermined dosimetric parameters. Needle insertion and implantation were done by urologists. As needed, modifications to the plan can be made, and the software recalculates the dose–volume histograms and isodose lines by using a real-time intraoperative dosimetry technique [7]. Patients were assigned to receive loose or intraoperatively built custom-linked (IBCL) seed brachytherapy based on the week of the month, with loose or IBCL seeds used during alternate weeks. In the present study, 14 and 15 patients were implanted with loose and IBCL seeds, respectively. Loose seeds were implanted using a Mick applicator (Mick Radio Nuclear Instruments, Mount Vernon, NY, USA). IBCL seeds were constructed using a Quicklink device (CR Bard, Covington, GA, USA) and implanted. Zauls et al. described the detailed mechanisms of constructing IBCL seeds [33], and we applied the same devices in our study. Immediately after all seeds had been implanted, intraoperative samples of peripheral blood were drawn from each patient.

### 4.4. CTC Detection

Twenty milliliters of peripheral blood was collected into 10 mL CellSave Preservation Tubes (Immunicon, Hatboro, PA, USA), which contained ethylenediaminetetraacetic acid (EDTA) as an anticoagulant and a cellular preservative. Samples were maintained at room temperature and processed within 72 h of collection. Blood samples of 7.5 mL were analyzed for CTCs.

The CellSearch system was used for the isolation and enumeration of CTCs. This system consists of the CellTracks AutoPrep and the CellTracks Analyzer II unit (Veridex LLC, Raritan, NJ, USA). The AutoPrep is a semiautomated sample preparation for the isolation of CTCs. The procedure enriches the sample for cells expressing epithelial cell adhesion molecule (EpCAM) using antibody-coated magnetic beads. After the magnetic separation, these cells were stained with the fluorescent nucleic acid dye 4′,6-diamidino-2-phenylindole (DAPI) and fluorescently labeled with anticytokeratin 8,18,19-phycoerythrin peridinin and anti-CD45 chlorophyll protein to distinguish epithelial cells from leukocytes. The stained and fluorescently labeled cells were analyzed for the identification of CTCs using the Analyzer II (Veridex LLC). The criteria for CTC included positive staining for DAPI and the cytokeratin and negative staining for the CD45. A CTC must show round or oval morphology.

### 4.5. Statistical Analysis

The primary outcome measures were changes in CTC detection rates from preoperative to intraoperative blood samples. As secondary outcome measures, incidence of increase in the intraoperative CTC count relative to the preoperative one was tested for association with clinicopathological and perioperative variables. For the purpose of analysis, clinicopathological and perioperative variables including age (>70 vs. ≤70), PSA at diagnosis (≥10 ng/mL vs. <10 ng/mL), Gleason score (≥8 vs. ≤7), clinical T stage (T1c–2c vs. T3a–4), biopsy positive core rates (>34% vs. ≤34%), prostate volume at brachytherapy (>25 cc vs. ≤25 cc), and NCCN risk criteria 2015 (high/very high vs. intermediate/low) were evaluated as dichotomous variables. A Fisher’s exact test was used to evaluate the primary and secondary outcome measures.

Sample size calculations determined that 60 patients would be needed to detect a 15% rise from preoperative to intraoperative CTC detection rates with α equal to 0.05 and power equal to 80%. A rise from preoperative to intraoperative CTC detection rates was estimated from the first 30 cases in this study.

Differences were regarded as statistically significant at the *p* < 0.05 level. Analyses were performed using SPSS, version 11.0 for Windows (SPSS, Inc., Chicago, IL, USA) and Microsoft Excel (Microsoft, Redmond, WA, USA).

## 5. Conclusions

We detected CTCs from samples immediately after insertion of needles in patients undergoing prostate brachytherapy for clinically nonmetastatic prostate cancer. Further research is needed to assess whether those cancer cells actually can survive and proliferate at distant sites. Although the brachytherapy approaches have demonstrated favorable long-term outcomes [6], understanding the mechanism of relapse should lead to the better treatment outcomes in patients undergoing prostate brachytherapy.

## Figures and Tables

**Table 1:** Patient characteristics (*n* = 59).

Factors	HDR (*n* = 30)		LDR (*n* = 29)		Total (*n* = 59)	
	Median	(Range)	Median	(Range)	Median	(Range)
Age (year)	71.5	(58–82)	70	(51–77)	71	(51–82)
PSA at diagnosis (ng/mL)	26.8	(4.5–396)	6.5	(4.2–14.1)	10.1	(4.2–396)
Prostate volume (cc) *	14.4	(4.6–29.7)	30.7	(20.3–58.4)	22.2	(4.6–58.4)
Number of needles	18	(18–18)	21	(17–29)	–	–
Duration of NHT (months)	16	(7–25)	0	(0)	–	–
	***n***	**(%)**	***n***	**(%)**	***n***	**(%)**
**Gleason Score**						
≤6	0	(0)	8	(28)	8	(14)
7	7	(23)	19	(65)	26	(44)
8 to 10	23	(77)	2	(7)	25	(42)
**Clinical T Stage**						
1c–2a	6	(20)	20	(69)	26	(44)
2b–2c	6	(20)	9	(31)	15	(25)
3a	11	(37)	0	(0)	11	(19)
3b	6	(20)	0	(0)	6	(10)
4	1	(3)	0	(0)	1	(2)
	***n***	**(%)**	***n***	**(%)**	***n***	**(%)**
**Biopsy Positive Core Rate**						
<34%	8	(27)	21	(73)	29	(49)
34%–67%	12	(40)	7	(24)	19	(32)
>67%	10	(33)	1	(3)	11	(19)
**NCCN Risk Criteria (2015)**						
Low	0	(0)	6	(21)	6	(10)
Intermediate	0	(0)	21	(72)	21	(36)
High	20	(67)	2	(7)	22	(37)
Very high	10	(33)	0	(0)	10	(17)

* Prostate volume was measured by transrectal ultrasound sonography immediately before insertion of needles. HDR: high-dose-rate brachytherapy; LDR: low-dose-rate brachytherapy; PSA: prostate-specific antigen; NHT: neoadjuvant hormonal therapy; NCCN: National Comprehensive Cancer Network.

**Table 2:** Characteristics of seven patients who changed to positive status for intraoperative circulating tumor cells (CTCs).

Type of Brachytherapy	HDR	HDR	HDR	HDR	LDR	LDR	LDR
Case number	9	26	34	36	8	19	43
Number of CTC counts (/7.5 mL)	2	1	1	1	1	1	1
Age (years)	71	75	65	75	58	65	67
Duration of NHT (months)	17	16	16	17	0	0	0
PSA nadir during NHT (ng/mL)	0.014	<0.008	<0.008	0.14	–	–	–
PSA at diagnosis (ng/mL)	31	13.5	17.6	66.7	8.6	4.6	14.1
Prostate volume (cc) *	7	29.7	21.3	13.9	26.1	38.4	37
Number of needles	18	18	18	18	24	28	18
Gleason score	8	8	7	9	6	7	7
Clinical T stage	1c	3a	3b	2c	2a	2a	2c
Biopsy positive core rate (%)	75	50	25	100	10	16.6	33.3
NCCN risk criteria 2015	H	H	VH	H	L	I	I

* Prostate volume was measured by transrectal ultrasound sonography immediately before insertion of needles; HDR: high-dose-rate brachytherapy; LDR: low-dose-rate brachytherapy; NHT: neoadjuvant hormonal therapy; PSA: prostate-specific antigen; NCCN: National Comprehensive Cancer Network; H: high risk; VH: very high risk; L: low risk; I: intermediate risk.

**Table 3:** Association of positive status for intraoperative circulating tumor cells (CTCs) with perioperative features (*n* = 59).

Factors	CTC Positive Rates	(*n*)	*p*
Age (>70 vs. ≤70 years)	8.8% vs. 16.0%	(3/34 vs. 4/25)	0.442
Type of brachytherapy (HDR vs. LDR)	13.3% vs. 10.3%	(4/30 vs. 3/29)	>0.999
NHT (yes vs. no)	13.3% vs. 10.3%	(4/30 vs. 3/29)	>0.999
PSA at diagnosis (≥10 vs. <10 ng/mL)	16.1% vs. 7.1%	(5/31 vs. 2/28)	0.424
Prostate volume (cc)	14.8% vs. 9.3%	(4/27 vs. 3/32)	0.691
Prostate volume/number of needle (≥1 vs. <1 cc/needle)	14.2% vs. 8.3%	(5/35 vs. 2/24)	0.689
Gleason score (≥8 vs. <8)	12.0% vs. 11.7%	(3/25 vs. 4/34)	>0.999
Clinical T stage (≥3a vs. ≤2c)	11.7% vs. 11.9%	(2/17 vs. 5/42)	>0.999
Biopsy positive core rate (>34% vs. ≤34%)	10.0% vs. 13.7%	(3/30 vs. 4/29)	0.706
NCCN risk criteria 2015 (H or VH vs. I or L)	12.5% vs. 11.1%	(4/32 vs. 3/27)	>0.999

## Abbreviations

LDR	low-dose-rate
HDR	high-dose-rate
CTC	circulating tumor cell
PSA	prostate-specific antigen
NCCN	National Comprehensive Cancer Network
TURP	transurethral resection of prostate
PSMA	prostate-specific membrane antigen
ADT	androgen deprivation therapy
IBCL	intraoperatively built custom-linked
EpCAM	expressing epithelial cell adhesion molecule
DAPI	4′,6-diamidino-2-phenylindole
